# Legionellosis on the rise: A scoping review of sporadic, community-acquired incidence in the United States

**DOI:** 10.1017/S0950268823001206

**Published:** 2023-07-28

**Authors:** Michelle A. Moffa, Clare Rock, Panagis Galiatsatos, Shantini D. Gamage, Kellogg J. Schwab, Natalie G. Exum

**Affiliations:** 1 Department of Medicine, Johns Hopkins University School of Medicine, Baltimore, MD, USA; 2 Division of Infectious Diseases, Department of Medicine, Johns Hopkins University School of Medicine, Baltimore, MD, USA; 3 Department of Hospital Epidemiology and Infection Control, Johns Hopkins Hospital, Baltimore, MD, USA; 4 Medicine for the Greater Good, Johns Hopkins University School of Medicine, Baltimore, MD, USA; 5 Division of Pulmonary and Critical Care Medicine, Johns Hopkins University School of Medicine, Baltimore, MD, USA; 6 U.S. Department of Veterans Affairs, National Infectious Diseases Service, Veterans Health Administration, Washington, DC, USA; 7 Division of Infectious Diseases, Department of Internal Medicine, University of Cincinnati College of Medicine, Cincinnati, OH, USA; 8 Department of Environmental Health and Engineering, Johns Hopkins Bloomberg School of Public Health, Baltimore, MD, USA

**Keywords:** built environment, climate, community-acquired pneumonia, drinking water, Legionella, Legionellosis (Pontiac fever), Legionnaires’ Disease

## Abstract

Over the past two decades, the incidence of legionellosis has been steadily increasing in the United States though there is noclear explanation for the main factors driving the increase. While legionellosis is the leading cause of waterborne outbreaks in the US, most cases are sporadic and acquired in community settings where the environmental source is never identified. This scoping review aimed to summarise the drivers of infections in the USA and determine the magnitude of impact each potential driver may have. A total of 1,738 titles were screened, and 18 articles were identified that met the inclusion criteria. Strong evidence was found for precipitation as a major driver, and both temperature and relative humidity were found to be moderate drivers of incidence. Increased testing and improved diagnostic methods were classified as moderate drivers, and the ageing U.S. population was a minor driver of increasing incidence. Racial and socioeconomic inequities and water and housing infrastructure were found to be potential factors explaining the increasing incidence though they were largely understudied in the context of non-outbreak cases. Understanding the complex relationships between environmental, infrastructure, and population factors driving legionellosis incidence is important to optimise mitigation strategies and public policy.

## Introduction

Legionnaires’ disease (LD), a type of pneumonia that is often severe and has a 7–10% mortality rate, is the leading cause of drinking water disease outbreaks in the United States (USA) [1–3]. Legionellosis, caused by infection with *Legionella* bacteria, includes both LD and the flu-like illness, Pontiac fever. LD is especially severe in older people, smokers, and those with compromised immune systems [1]. Infections are caused by the inhalation of waterborne *Legionella* from aerosolised sources in the built environment such as showers, sinks, and cooling towers. Healthcare-associated legionellosis infections and travel-associated infections each account for about 20% of cases in the USA, while the remaining estimated 60–65% of cases are considered community-acquired [2, 3]. National analyses suggest that up to 95% of community-acquired legionellosis cases in the USA are sporadic, meaning that they are not associated with a known outbreak, cluster, or identifiable environmental exposure [3, 4].

Reporting to the Centers for Disease Control and Prevention (CDC) National Notifiable Diseases Surveillance Systems (NNDSS) since 2002 shows that age-standardised legionellosis incidence has been increasing at an average annual rate of 9.3% [2]. The annual increase in reported legionellosis cases is highest in the Middle Atlantic and Midwest regions [4]. An investigation of the true LD burden in the USA indicates that the current observed incidence rate of 1.2 cases per 100,000 population is substantially lower than the estimated rate of 11.6 cases per 100,000 population [4, 5].

There is a lack of epidemiological data on the underlying drivers of increasing legionellosis incidence in the USA [2]. Numerous clinical and environmental factors have been hypothesised for the year-on-year increase. There is a need to differentiate between whether there is a true increase in cases or whether the increase in reported incidence is an artefact of more successful detection. Potential clinical factors include improved clinical case capture due to more accessible diagnostic tools, increased physician awareness of atypical pathogens of community-acquired pneumonia (CAP), and improved reporting practices to the CDC [6, 7]. The increase in incidence may also be due to demographic factors such as an ageing and increasingly immunocompromised population [8, 9]. Environmental drivers causing *Legionella* to be more prevalent in water systems and increasingly exposing the population may include drinking water infrastructure factors [10, 11] and climate factors such as changing temperature and precipitation [12].

In this scoping review, we aimed to synthesise evidence from across the literature regarding the drivers of sporadic, community-acquired legionellosis cases in the USA since 2006. The weight of evidence from the scoping review is then discussed to determine the impact of these factors as drivers of increasing incidence.

## Methods

We conducted a scoping review to identify studies and reviews that discuss the following themes: (i) epidemiological trends and potential drivers of increasing sporadic, community-acquired legionellosis incidence and (ii) trends in clinician awareness, diagnostic testing, and reporting of legionellosis. We focused the search on studies within the USA due to the complexity of factors influencing legionellosis transmission and the major differences in population demographics, water treatment and management, and public policy between the USA and other countries. We include international studies in the discussion to help interpret and contextualise our findings.

Peer-reviewed literature in PubMed was searched in November 2022 using the following keywords: (“Legionnaires’ Disease” OR “legionellosis” OR “*Legionella*” OR “*Legionella pneumophila”* OR “Pontiac Fever”) AND (“incidence” OR “case count” OR “burden” OR “prevalence” OR “diagnos*” OR “reporting” OR “surveillance” OR “trend”). We followed the PRISMA Extension for Scoping Reviews while reporting our findings as applicable [13] and acknowledge that the evidence presented in this scoping review is not considered exhaustive given that systematic methods were not used to select studies.

We included only studies written in English that discussed one or both of the themes above. To focus on understanding national and regional incidence trends of sporadic, community-acquired disease, we excluded individual case reports, outbreak reports, and water monitoring reports without the inclusion of epidemiological data. We also excluded studies that exclusively discussed the following: nosocomial or travel-associated legionellosis; novel clinical diagnostic methods; microbiological studies; and legionellosis treatment, prevention, or control strategies. We restricted the search to papers published in 2006 or later, which is the first full calendar year after the CDC changed the legionellosis case definition in 2005 [2].

We screened 1,738 titles resulting from the PubMed search that may include relevant information. From abstracts and full text, we then identified 18 articles that met our inclusion criteria and had information or analysis relevant to our research question. The rating systems used for the magnitude of impact and quality of evidence to evaluate the articles as potential drivers are described in Table 1.

**Table 1.** Rating system to evaluate the potential drivers identified in articles of scoping review

Magnitude of impact of potential drivers	
Rating system	Description
Major	Strong effects and significant associations with analysis that examined increasing incidences over multiple years
Moderate	Significant associations between drivers and incidence with smaller effect sizes over time
Minor	Studies found small effect sizes and only examined incidence over limited time periods
Null effect	Associations lacked significance between the driver and increasing incidence
Insufficient evidence	Not enough evidence to classify a potential driver

**Quality of evidence of studies examined for a potential driver**	
**Rating system**	**Description**
High	Three or more national-scale USA-based studies that found significant associations between the driver and incidence rates over both time and geographic scope
Medium	One to two USA studies that captured temporal changes in incidence and not national in scope
Low	Studies identified were cross-sectional or their design was not able to determine the trend in incidence over time

## Results

Table 2 summarises the potential drivers of increasing legionellosis incidence identified in the scoping review and the ratings assigned. All 18 of the included papers present evidence relevant to the epidemiological trends and potential drivers of sporadic, community-acquired legionellosis incidence. Seven of the included papers additionally address trends in clinical awareness, diagnostic testing, and reporting of legionellosis. The included papers are described in greater detail in Supplementary Table S1.

**Table 2.** Potential drivers of increasing legionellosis incidence in the United States, 2006–2022: magnitude of impact and quality of evidence

Potential driver	Magnitude of impact (major, moderate, minor, null, insufficient evidence)	Quality of evidence (high, medium, low)	References
**Clinical case capture**			
CDC case definition changes	Null effect	Medium	Barskey et al. [2]
Improved reporting to the CDC	Null effect	Medium	Alarcon Falconi et al. [14]; Dooling et al. [15]
Increased clinician awareness	Insufficient evidence	Low	Alarcon Falconi et al. [14]; Barskey et al. [2]; Simmering et al. [16]
Increased testing and UAT usage	Moderate	Medium	Allgaier et al. [17]; Gamage et al. [18]; Schoonmaker-Bopp et al. [19]; Toberna et al. [20]
**Racial and socioeconomic inequities**			
Racial inequity	Insufficient evidence	Low	Barskey et al. [2]; Farnham et al. [21]; Hicks et al. [4]; Hunter et al. [22]
Socioeconomic inequity	Insufficient evidence	Low	Farnham et al. [21]; Gleason et al. [23]; Hunter et al. [22]
**Population susceptibility**			
Ageing population	Minor	High	Alarcon Falconi et al. [14]; Barskey et al. [2]; Han [24]; Hicks et al. [4]
Increase in underlying medical conditions	Insufficient evidence	Low	Cooley et al. [25]
**Climate and hydrometeorological factors**			
Precipitation	Major	High	Gleason et al. [26]; Han [24, 27]; Passer et al. [28]; Simmering et al. [16]
Temperature	Moderate	High	Gleason et al. [26]; Han [24, 27]; Passer et al. [28]
Relative humidity	Moderate	High	Gleason et al. [26]; Passer et al. [28]; Simmering et al. [16]
**Built environment**			
Water infrastructure	Insufficient evidence	Low	Cassell et al. [29]; Gleason et al. [23]
Older housing stock and urbanisation	Major	Low	Farnham et al. [21]; Gleason et al. [23]

### Clinical case capture

The following potential drivers of improved case capture were identified from the literature: i) more consistent reporting to the CDC; ii) an increase in clinician awareness; iii) more frequent diagnostic testing; and iv) increased access to urinary antigen tests [2, 4, 14, 16, 17, 19, 21].

#### Increased reporting

The CDC has revised the legionellosis case definition several times since reporting began in 1976 – most recently in 2005 and 2019 – but it is unlikely that these small changes have impacted reporting data [2]. Alarcon Falconi et al. [14] found a history of underreporting diagnosed cases to the CDC that decreased over time, suggesting improvement in reporting to the CDC. More recently, in 2011–2013, the CDC instituted an Active Bacterial Core Surveillance Programme at ten U.S. sites and found that legionellosis incidence was similar between passive NNDSS reports and the cases projected from active surveillance [15]. Underreporting to the CDC has likely decreased over time, and this potential driver may have a null effect on increasing incidence.

#### Increased clinician awareness

Reported cases in the USA remained relatively stable between 1990 and 2002 at an average of 1,268 cases annually and then increased sharply to 2,223 cases in 2003 [30]. This increase coincided with the severe acute respiratory syndrome (SARS) pandemic. Several articles suggested that clinician efforts to identify the aetiology of CAP cases may have increased during the SARS pandemic, thereby increasing the number of *Legionella* tests performed and cases of legionellosis identified, though there is minimal evidence to support this theory [2, 14, 16]. There remains insufficient evidence to understand how clinician awareness affected legionellosis surveillance in the USA during the 2003 SARS pandemic and what, if any, impact clinician awareness has played in the continuing incidence increase since then.

#### Increased testing and use of urinary antigen test diagnostics

The urinary antigen test (UAT) is a rapid, simple, and widely used diagnostic method compared with culturing *Legionella.* UATs were used to diagnose 69–70% of cases in 1998 [2, 14, 15], and they increased to over 90% by 2011 [15]. We identified two studies that included or discussed electronic health record (EHR) data from the USA showing how the frequency of legionellosis testing has changed over time. Allgaier et al. [17] analysed a national EHR database of adults hospitalised with a primary diagnosis of pneumonia across 177 hospitals. From 2010 to 2015, 26% of these patients were tested for *Legionella.* This percentage increased over time in the Midwest and Southern regions, and in the Northeast region, the percentage increased from approximately 30% in 2010 to 40% in 2015. Though an increasing percentage of hospitalised pneumonia patients were tested for *Legionella*, the underlying average annual test positivity rate over this same timeframe remained relatively constant at around 1.5%, indicating that increasing testing for *Legionella* is a driver of increasing incidence [17]. On a smaller scale, a study of UAT usage in a Wisconsin-based healthcare system found a relatively consistent number of tests that were ordered annually between 2013 and 2017 [20]. A cross-sectional study by Gamage et al. [18] analysed LD data in the national U.S. Veterans Health Administration from 2014 to 2016 and found that increased use of UAT regionally often results in lower test positivity. These studies present medium-quality evidence to support the idea that increased testing and improved diagnostics are a driver of rising legionellosis, with a moderate magnitude of impact on increasing incidence.

### Racial and socioeconomic inequities

Epidemiological studies identified in the scoping review frequently found racial and socioeconomic disparities in legionellosis incidence in the USA [2, 4, 15, 17, 20–23]. However, race and ethnicity data are often not reported with legionellosis case data across many states in the USA [2, 16], making it challenging to understand the role that racial inequities contribute to increasing incidence.

#### Racial inequity

Many analyses of legionellosis in the USA show incidence is highest among Black or African American individuals relative to other racial groups [2, 4, 15, 17, 21, 22]. In an analysis of cases reported to the CDC, Barskey et al. [2] found that when comparing a 1992–2002 baseline to 2018 data, the age-standardised incidence has increased at twice the rate among Black people (from 0.47/100,000 population in 1992–2002 to 5.21/100,000 population in 2018) relative to White people (from 0.37/100,000 population to 1.99/100,000 population). In New York City, legionellosis incidence from 2002 to 2011 was higher among the non-Hispanic Black population, at 2.15/100,000 population compared with 1.56/100,000 population for non-Hispanic Whites, though the difference was not statistically significant [21]. Many national epidemiological studies do not stratify data specifically for Hispanic/Latino people due to missing ethnicity data for over 30% of cases reported to the CDC [2, 4]. Despite these important racial disparities, we concluded that major data gaps and a lack of analysis linking any temporal or causal component of structural racism to legionellosis trends prevent us from determining the magnitude of impact that racial inequities have on increasing incidence.

#### Socioeconomic inequity

Socioeconomic demographics have shown significant relationships within multiple state-based studies in the USA [20, 21, 23]. Income was the most studied social determinant of health identified in a narrative review of disparities in LD incidence [22]. In New York City, the yearly age-adjusted incidence of community-acquired legionellosis between 2002 and 2011 was 2.5 times higher in the census tract with the highest poverty level (3/100,000 population) compared with the census tract with the lowest poverty level (1.2/100,000 population) [21]. In New Jersey, poverty level was the strongest risk factor for legionellosis in multivariate models using various census tract-level sociodemographic variables. High-risk census tracts were also more likely to have lower education levels [23]. Given that none of these studies were national in scale or discussed how poverty dynamics may be changing over time, there was insufficient evidence about the magnitude of impact that socioeconomic inequity may have on increasing incidence.

### Population susceptibility

Numerous epidemiological studies have established that legionellosis risk increases with age, smoking, and comorbidities such as chronic obstructive pulmonary disease (COPD), diabetes, kidney disease, and immunosuppression [25].

#### Ageing population

An ageing population is frequently cited as a contributing factor to increasing legionellosis incidence in the USA [2, 4, 5, 14, 24]. Overall, the age distribution of legionellosis cases reported to the CDC has remained relatively constant over time [14]. Three longitudinal epidemiological studies spanning from the 1990s through 2018 found that age-standardised incidence in the USA has increased at a slightly lower rate than crude, unadjusted incidence [2, 4, 24], indicating that an ageing population may have a minor impact on increasing incidence, backed up by high-quality evidence.

#### Increase in underlying medical conditions

None of the studies identified for the scoping review analysed how medical conditions or lifestyle factors (e.g. smoking) associated with LD risk have changed over time. Therefore, there was insufficient evidence to evaluate their impact on legionellosis incidence trends.

### Climate and hydrometeorological factors

Climate change is expected to have a promotive effect on waterborne infectious diseases such as legionellosis. The consistent seasonality observed with legionellosis incidence in the USA points to environmental factors as important potential drivers of disease. We identified five studies that examined longitudinal legionellosis case data with hydrometeorological factors to understand the impact of weather and climate on increasing incidence [16, 24, 26–28].

#### Precipitation

With robust multivariate regression modelling, there was a consistently significant promotive effect of increased precipitation on the risk of disease [16, 26, 28]. Gleason et al. [26] found a strong positive association between precipitation and increased legionellosis rates in New Jersey for the monthly, but not daily, precipitation variable, indicating that an extended latency period may be required to have a promotive effect on *Legionella* proliferation in the environment. Similarly, in Passer et al. [28], when lag effects were introduced to create a 14-day lagged average precipitation variable, a strong effect was found for the increase in the risk of sporadic infection. In Simmering et al. [16], a case–control study design found that there was nearly 20 mm more rain (80.4 mm) for cases than for controls (61.7 mm). These studies give confidence to the classification that precipitation is a major driver of sporadic, community-acquired legionellosis infections, backed up by high-quality evidence.

#### Temperature

Temperature was found to have a promotive effect on legionellosis incidence rates in 33 Eastern U.S. states and the District of Columbia [27]. A state-wide time-series study in New Jersey found that temperature was less predictive of legionellosis risk than wet, humid weather though a positive association between temperature and disease risk was found in a univariate model [26]. Han [24] found that mean temperature had a promotive effect on legionellosis incidence when the analysis was restricted to data in the peak season of May to October. Passer et al. [28] found an increased risk of sporadic infections when lag effects were introduced to create a 14-day lagged average temperature variable. Overall, there was high-quality evidence to classify temperature as having a moderate impact as a driver of increasing legionellosis incidence.

#### Relative humidity

Relative humidity (RH) was found to be positively associated with an increased risk of legionellosis in numerous studies. In Simmering et al. [16], regardless of precipitation, warm (60–80 °F) and humid weather was a major risk factor for disease, especially in the very humid months (RH >80%). Gleason et al. [26] used a time-series design to show that average RH was positively associated with disease risk. Passer et al. [28] found a positive association for RH in a fully adjusted model though the effect was weaker than the effect of precipitation. The quality of evidence presented was considered high to classify RH as moderate for its magnitude of impact.

### Built environment

Few articles analysed the relationship between the built environment and legionellosis incidence in the context of sporadic, community-acquired infections.

#### Water infrastructure

Two state-level spatial analyses of legionellosis incidence included water source variables. In Connecticut, the incidence was higher in regions with higher rates of private well water usage, with presumably no disinfectant residuals to limit microbial growth in pipes [29]. In New Jersey, there was no statistically significant association between LD incidence and whether the public drinking water source was groundwater versus surface water [23]. Given the lack of epidemiological nationwide analyses or analyses of specific water infrastructure characteristics, there was insufficient evidence to classify its magnitude of impact on incidence.

#### Older housing stock and urbanisation

Several studies found that legionellosis incidence is higher in urban environments, but there was limited discussion about mechanisms driving incidence in urban contexts [17, 28, 29]. In a spatial analysis of census tracts in New Jersey, Gleason et al. [23] found that having a greater percentage of housing units built pre-1970 was strongly associated with legionellosis incidence; the effect was even stronger with pre-1950 housing. Higher percentages of vacant housing and renter-occupied housing were also associated with higher incidence, though the latter relationship did not hold when controlling for poverty and pre-1950 housing [23]. Populations living in higher-poverty neighbourhoods in New York City were also found to have an increased risk of acquiring legionellosis, indicating that neighbourhood-level factors need to be examined in greater depth [21]. The strength of association found in Gleason et al. [23] regarding older housing stock classified this potential driver as having a major impact on increasing incidence though the quality of evidence was low given the singular geographically limited study on the topic.

## Discussion

This scoping review assessed the available evidence for legionellosis risk factors and potential differing impacts on driving the increase in incidence observed across the USA since 2006. Although there is a lack of strong evidence to explain the trend of increasing incidence, the included literature suggests that it represents a true increase in disease and cannot be solely explained by improved clinical case capture. Numerous clinical, environmental, infrastructure, and demographic factors are likely playing interconnected roles in driving legionellosis incidence.

### Clinical case capture

While improved reporting and diagnostic testing in the early 2000s likely contributed to the rise in reported legionellosis cases in that period, this does not explain the more recent rise in incidence. Between 2002 and 2018, the largest increase in age-standardised incidence occurred in 2016–2018, which cannot be explained by increased clinical testing and reporting trends [2]. In the VA healthcare system with extensive testing for LD in patients that may be more susceptible to infection, increased use of UAT regionally often results in lower test positivity [18]. The increase in testing volume may be due to changes in clinician awareness, health-seeking behaviour, or other factors. Though official clinical practice guidelines about CAP diagnosis by ATS/IDSA recommend *Legionella* testing only in adult patients with severe CAP or when there are epidemiological indicators such as recent travel or a *Legionella* outbreak, implementation of the guidelines may miss over 40% of legionellosis cases [31, 32].

While included analyses of EHR data from the USA do not give a clear picture of the impact of increased testing incidence, two international analyses present convincing evidence that incidence is increasing at a faster rate than testing [33, 34]. An epidemiological study of LD in Hong Kong found that between 2010 and 2015, UAT frequency increased by 127% and LD case frequency by 230% [33]. Similarly, an epidemiological study of community-acquired *Legionella* infections in a Spanish hospital found that the number of UAT orders increased 3.4 times and case incidence increased 5.9 times when comparing 2001–2005 with 2011–2015 data [34]. Further, a qualitative study about Swiss physicians’ approach to CAP diagnosis concluded that awareness about LD is generally high and that changing awareness does not appear to explain increasing incidence trends in Switzerland [35]. Because these international studies may not be generalisable to U.S. health care, there is a need for future quality analyses of EHR data and clinician knowledge, attitudes, and practices towards *Legionella* testing to better understand clinical trends in the USA. Reported legionellosis incidence may increase in future decades as diagnostic methods such as PCR – which can detect species and serogroups other than *L. pneumophila* serogroup 1 – become more widespread. This shift was demonstrated in a New Zealand analysis that found a marked increase in incidence corresponding with the uptake of molecular diagnostic methods [36]; future studies in the USA will need to account for the impact of these changes.

### Racial and socioeconomic inequalities and population susceptibility

There is insufficient evidence in the reviewed literature to characterise the impact of racial and socioeconomic inequities on increasing legionellosis incidence in the USA. The high proportion of legionellosis cases reported to the CDC with unknown race – nearly one-fifth of cases from 2000 to 2009 – limits our ability to fully understand the role race plays in risk [4]. It is evident that factors driving increased risk are disproportionately affecting Black people. The racial disparity has been suggested to be largely driven by economic differences [2, 21]. Disparities in housing stock or community-level infrastructure such as proximity to industrial buildings and cooling towers, ageing water infrastructure and premise plumbing, and the percentage of vacant buildings may drive racial and socioeconomic inequities in environmental exposures [15, 20, 21]. Racial inequities in access to preventive healthcare and higher rates of underlying medical conditions may also contribute to legionellosis as a health disparity issue [2, 22]. Although there is a lack of evidence about how these dynamics are changing over time with respect to legionellosis, health equity research has found that both racial and socioeconomic health inequities in the USA have persisted [37, 38]. Our review demonstrates an imperative for further examination of the interaction of legionellosis incidence with health disparities and population susceptibility to better implement equitable interventions.

### Climate and hydrometeorological factors

We found that climate and hydrometeorological factors, notably precipitation, underlying the seasonality of LD are major drivers of increasing incidence. Temperature and RH were found to have more moderate impacts with high quality of evidence. The relationships between these climate factors and legionellosis were strengthened when analyses were restricted to the Midwest and Northeast regions of the USA [27]. This finding raises concerns that climate change will continue to increase legionellosis incidence in the USA under current trends of more frequent days with temperatures above 90 °F and increased precipitation [39]. Numerous international studies have similar findings that increased precipitation [40–43], elevated temperature [40–42, 44, 45], and higher relative humidity [41–43, 46] are associated with increased community-acquired legionellosis cases and clusters. Future research is needed to understand the exposure pathways between changing hydrometeorological factors and LD infections.

### Built environment

Our review did not identify any studies that examined deficiencies in drinking water systems and their impacts on sporadic, non-outbreak legionellosis incidence. A narrative review of social determinants of health and legionellosis similarly concluded that this relationship has been understudied for sporadic, community-acquired cases [22]. At a county level, Rhoads et al. [47] analysed an outbreak of legionellosis cases in the context of a source water switch that depleted disinfectant residual in the piped network in Flint, Michigan. Low levels of residual chlorine in municipal water systems, changes in corrosion control, and increases in source water turbidity have similarly been linked to legionellosis clusters [48, 49]. These studies were excluded from our review because they were conducted in the context of outbreaks, but they highlight the impact that drinking water supplies can have on LD incidence. In a national analysis, Holsinger et al. [50] reported legionellosis outbreaks linked to buildings served by public water systems and concluded most outbreaks linked back to healthcare and hotel buildings and buildings served by large (>10,000 people) water systems. The authors did not analyse sporadic cases as they noted that source attribution is lower for such cases [50]. We identified a single study attributing legionellosis incidence to older housing units in New Jersey, which may be due to ageing community water infrastructure or housing units’ complex water systems [23]. Future epidemiological research should examine correlations between sporadic, community-acquired incidence and potentially relevant water system characteristics such as source water type and quality, disinfection type and level, utility age and maintenance, and water main breaks [16, 47, 51]. There is also a need for future investigation to understand the risk factors that cause *Legionella* from public water systems to colonise and proliferate in the plumbing of single-family residential homes or other buildings [51] and how these built environment risk factors may be changing over time.

### Limitations

The scoping review is limited by the small number of peer-reviewed articles examining the epidemiology of sporadic, community-acquired legionellosis. Outbreak cases represent a smaller proportion of cases in the USA [3, 4] but constitute a large amount of legionellosis literature, thereby leading to conclusions about the drivers of increasing incidence that may not be relevant to sporadic, community-acquired legionellosis. The review also excluded papers that solely addressed travel-associated or nosocomial cases though many of the included papers do not differentiate between these categories. Although we searched for both Legionnaires’ disease and Pontiac fever, we did not identify any papers about Pontiac fever that fit our inclusion criteria.

## Summary

This review synthesised and assessed the available evidence about factors that may be driving the increasing incidence of sporadic, community-acquired legionellosis in the USA since 2006. Given the large number of studies screened and the comparatively small number of articles identified that met the inclusion criteria, more work is needed to understand the mechanisms that underlie the more than fivefold increase in reported legionellosis cases in the past two decades, amounting to approximately 10,000 cases in 2018 [2]. This review found clear evidence for climate change as one of these major drivers of increasing incidence. Future research should investigate exposure pathways from the built environment and how racial and socioeconomic inequities are impacting disease incidence. A better understanding of the major factors driving the increase in sporadic, community-associated legionellosis incidence in the USA would inform targets for improved preventive measures and assist in optimising public policy.

## Supporting information

Moffa et al. supplementary material Moffa et al. supplementary material

## Data Availability

Readers may replicate the findings in this scoping review by conducting the same search of peer-reviewed literature in PubMed using November 2022 as the end date and the same keywords described in the Methods section.

## Supplementary material

For supplementary material accompanying this paper visit http://doi.org/10.1017/S0950268823001206. click here to view supplementary material
