# Supplementary material for: Legionellosis on the rise: A scoping review of sporadic, community-acquired incidence in the United States
Source: Epidemiol Infect. 2023 Jul 28;151:e133. doi: 10.1017/S0950268823001206 (PMC10540183; doi:10.1017/S0950268823001206)
Supplement: Moffa et al. supplementary material [file S0950268823001206sup001.docx]

**Table S1 List of included studies and drivers of legionellosis incidence examined in each study.**

| Reference | Driver(s) examined | Study location | Study type and methods | Years studied |
| --- | --- | --- | --- | --- |
| Alarcon Falconi et al., 2018 | Clinical case capture;  Aging population | United States | Retrospective observational analysis of national data to assess seasonality of legionellosis | 1993-2015 |
| Allgaier et al., 2021 | Clinical case capture | United States | Retrospective cohort analysis of national EHR data to understand testing rates and patient risk factors | 2010-2015 |
| Barskey et al., 2022 | Clinical case capture;  Inequities;  Aging population | United States | Retrospective observational analysis of national data to understand epidemiologic patterns | 1992-2018 |
| Cassell et al., 2018 | Water infrastructure | Connecticut | Retrospective spatial models and time series methods to analyze association between sporadic legionellosis and natural aquatic environment, including comparing municipal and private well water supplies | 1999-2015 |
| Cooley et al., 2020 | Underlying medical conditions | United States | Retrospective observational analysis of active surveillance data and national survey data to characterize clinical risk factors | 2011-2015 |
| Dooling et al., 2015 | Clinical case capture | United States | Retrospective observational analysis comparing active surveillance data from 10 sites with national passive surveillance data | 2011-2013 |
| Farnham et al., 2014 | Inequities;  Housing and urbanization | New York, New York | Retrospective observational analysis of case records to understand epidemiology and risk factors | 2002-2011 |
| Gamage et al., 2018 | Clinical case capture | United States | Cross-sectional analysis of national Veterans Affairs data to understand burden of disease and exposures | 2014-2016 |
| Gleason et al., 2016 | Climate and hydrometeorological factors | United States | Retrospective time series and case-crossover methods to evaluate association of legionellosis and meteorological factors | 2003-2013 |
| Gleason et al., 2017 | Inequities;  Water infrastructure;  Housing and urbanization | New Jersey | Retrospective spatial and geovisual methods to compare legionellosis risk across census tracts and understand population-level determinants | 2003-2013 |
| Han, 2019 | Climate and hydrometeorological factors | United States | Retrospective observational analysis of national data to understand environmental effects on incidence rates | 2014-2016 |
| Han, 2021 | Aging population;  Climate and hydrometeorological factors | United States | Retrospective observational analysis of national data to understand environmental effects on incidence rates | 1999-2018 |
| Hicks et al., 2011 | Inequities;  Aging population | United States | Retrospective observational analysis of national data | 2000-2009 |
| Hunter et al., 2021 | Inequities | United States | Narrative review of articles that focus on social determinants of health and LD incidence | 1979-2019 |
| Passer et al., 2020 | Climate and hydrometeorological factors | Minnesota | Retrospective ecological analysis of state surveillance data and environmental factors | 2011-2018 |
| Schoonmaker-Bopp et al., 2021 | Clinical case capture | New York | Retrospective observational analysis of state testing and investigation data | 1978-2017 |
| Simmering et al., 2017 | Clinical case capture;  Climate and hydrometeorological factors | United States | Retrospective observational analysis of national hospitalization data to understand effects of environmental factors on risk | 1998-2011 |
| Toberna et al., 2020 | Clinical case capture | Wisconsin | Descriptive ecologic analysis of hospital EHR data to understand urinary antigen testing and geodemographic patterns | 2013-2017 |
